# Reassessment of peripheral nerve stimulation thresholds for the Impulse model‐optimized asymmetric head gradient coil

**DOI:** 10.1002/mrm.30523

**Published:** 2025-05-23

**Authors:** David A. Feinberg, Samantha J. Ma, Erica Walker, Alexander J. S. Beckett, Dominik Rattenbacher, Elmar Rummert, Peter Dietz, Mathias Davids, Nicolas Boulant

**Affiliations:** ^1^ Department of Neuroscience and Helen Wills Neuroscience Institute, Brain Imaging Center University of California Berkeley California USA; ^2^ Advanced MRI Technologies Sebastopol California USA; ^3^ Siemens Medical Solutions Malvern Pennsylvania USA; ^4^ Siemens Healthcare Erlangen Germany; ^5^ A.A. Martinos Center for Biomedical Imaging, Department of Radiology Massachusetts General Hospital Charlestown Massachusetts USA; ^6^ Harvard Medical School Boston Massachusetts USA; ^7^ University of Paris‐Saclay, CEA, CNRS BAOBAB, NeuroSpin Gif sur Yvette France

**Keywords:** brain imaging, high performance gradient, magneto‐stimulation thresholds, NexGen 7 T, peripheral nerve stimulation

## Abstract

**Purpose:**

Peripheral nerve stimulation (PNS) remains a physiologic limitation to boosting spatiotemporal resolution with more powerful gradients. We investigate discrepancies in previous measurements and model predictions from PNS experienced by volunteers scanned with the investigational “Impulse” gradient coil on the NexGen 7T scanner.

**Methods:**

Twenty‐nine volunteers (18 males, mean ± standard deviation age 52.2 ± 17.1 years) underwent PNS characterizations in the scanner. The process was repeated after the subject positions were moved by 2 and 4 cm toward the feet, away from isocenter. These new data were compared with prior experimental data acquired at the factory (32 volunteers, 16 males, mean ± standard deviation age 58.3 ± 13.5 years) and to modeling results initially used to guide the gradient winding pattern.

**Results:**

The PNS threshold for the x‐axis (left–right) was significantly below the threshold level predicted by the model used to optimize the wiring pattern and thresholds measured in the factory, whereas there was closer agreement for the y‐axis (anterior–posterior) and z‐axis (superior–inferior). The x‐axis threshold increased as the subject was moved in the Z‐direction toward the foot end of the magnet, at the expense of gradient nonlinearity distortions. Sensitivity of the threshold for the x‐axis was measured as 20 mT/m per centimeter Z‐offset.

**Conclusion:**

The PNS threshold of the x‐axis measured in the scanner was much lower than predicted by the optimization model and as measured at the factory. Our measurements verified that PNS thresholds of asymmetric head gradient coils were sensitive to head position, subject variability, and age. The discrepancy of the PNS prediction model remains to be elucidated.

## 1 INTRODUCTION

The usable performance of magnetic gradient coils for human MRI is determined by hardware limitations and by physiological constraints—primarily peripheral nerve stimulation (PNS)^1^, ^2^, ^3^, ^4^, ^5^, ^6^, ^7^, ^8^, ^9^ and acoustic sound pressure noise.^10^ With these constraints, a gradient coil designed for head imaging can achieve higher performance than whole‐body gradient coils, given their smaller diameter with lower inductance coupled with their shorter length, which reduce both cardiac stimulations and PNS in the thorax and body.^1^, ^2^, ^4^, ^11^, ^12^

An investigational 7T MRI scanner was designed and built to achieve higher spatial resolution, signal‐to‐noise ratio, and acquisition speed in human brain imaging by redesigning several hardware subsystems including the gradients. The NexGen 7T scanner project included a novel high‐performance head gradient coil “Impulse,” the first 128‐channel receiver system at 7 T, and a 16‐channel radiofrequency (RF) transmission architecture.^13^ The gradient coil was integrated into a 7T scanner where higher forces from high amplitude gradients at high field are challenging. The gradient coil was also designed with a relatively large inner diameter of 44 cm to provide space for novel high‐density RF array coils and shim array around the human head. With 200‐mT/m maximum gradient amplitude and 900‐mT/m/ms slew rate,^1^, ^13^ the investigational Impulse gradient coil is currently the highest performance gradient operating in a human 7T scanner. The scanner was built to advance resolution in human brain imaging, particularly diffusion and functional MRI using echo‐planar imaging (EPI) sequences and derivative echo train sequences, for which spatiotemporal resolution is highly dependent on gradient performance: slew rate and maximum gradient amplitude. The scanner is based on the MAGNETOM Terra 7T scanner (Siemens Healthcare, Erlangen, Germany) with modifications designed and planned in a collaboration between UC Berkeley and Siemens.

**FIGURE 1:**
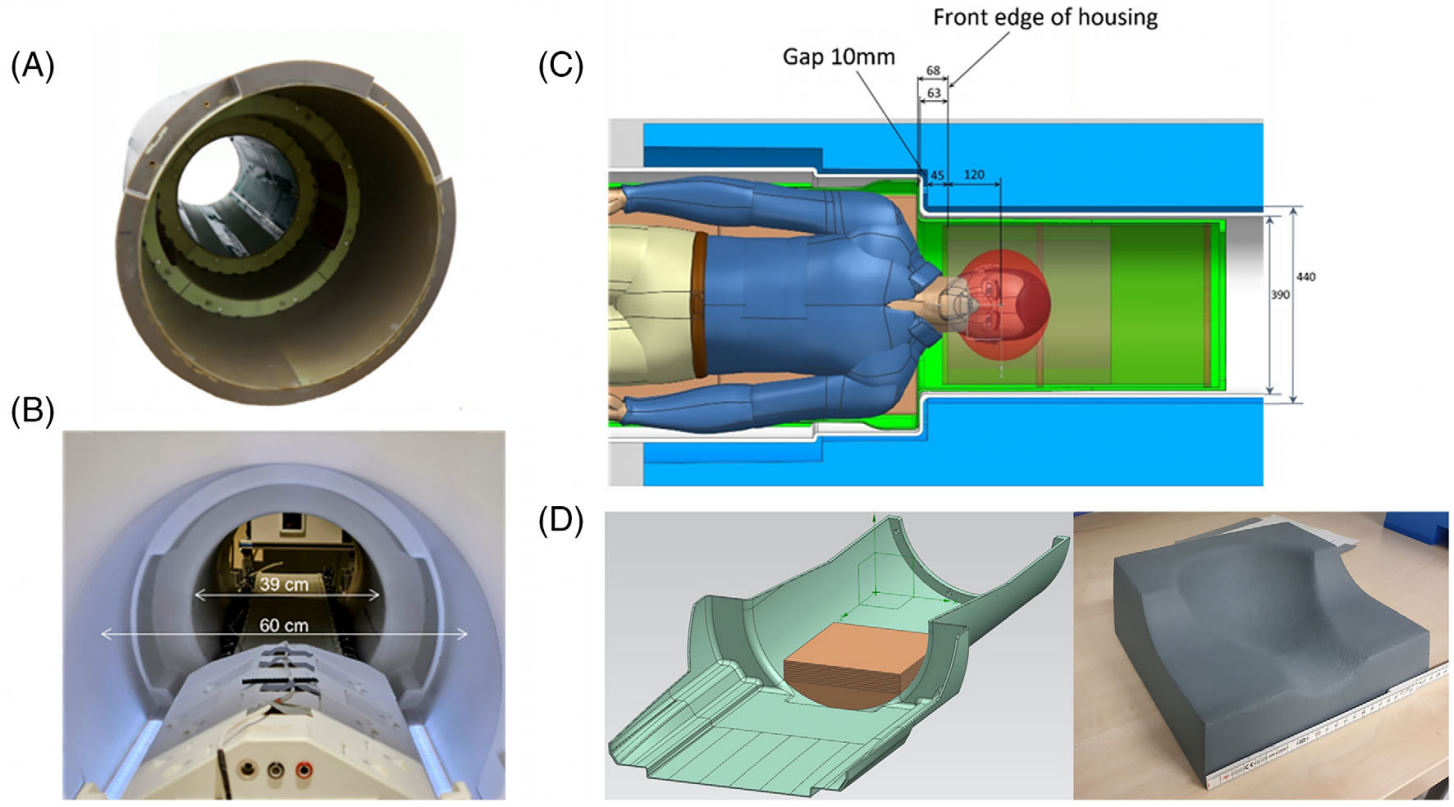
Impulse gradient coil design and testing setup. (A) Photo of Impulse gradient coil with three‐layer winding design and intermediate layer for optimization of torque and peripheral nerve stimulation threshold. (B) Photo of bore liner covering Impulse head gradient with vertical wall and shoulder cutouts. (C) CAD drawing showing the fitting of the bore liner (*white*) with respect to the gradient coil (*light blue*) and the patient table (*green*). Acoustic dampening material (not shown) fills the space between the bore liner and the gradient coil. (D) CAD drawing of patient table extension (*green*) to support subject's head at the factory using spacer plates (*brown*) and the gray foam head pad for subject positioning at isocenter.

The Impulse gradient coil has a novel three‐layer winding pattern instead of the conventional two‐layer shielded design^14^ with the intermediate winding layer available for optimizing PNS, torques, and forces (Figure 1A–C). Important to the topic of this report, the electromagnetic‐neurodynamic PNS modeling method^1^, ^11^, ^15^ was used to guide the design of the winding pattern of the intermediate coil layer, with the intention to raise PNS thresholds through balancing PNS hot spots between the shoulder and face. The levels of PNS balancing were chosen to increase the usable performance without raising thresholds beyond the hardware limit, resulting in a coil design without full PNS balancing. The PNS predictions were based on modeling electric fields induced by a given geometry in electromagnetic body models, followed by coupling of these electric fields to a model of the peripheral nervous system to predict quantitative PNS thresholds and stimulation sites. The PNS informed coil design workflow relied on designing a number of candidate coil designs with varying design parameters (e.g., coil former geometry, number of coil layers, field linearity, size of region of linearity), followed by a prediction of the PNS characteristics for each candidate design. The target, therefore, was a linear dependence of the magnetic field homogeneity on the z‐axis, the Bz field in the region of interest with trade‐offs with this constraint, by tuning the magnitude and orientation of the transverse, concomitant, magnetic fields^16^, ^17^ arising from Maxwell's equations and influencing the electric field distributions. The PNS predictions allowed for iterative adjustment of the coil design parameters to raise the worst‐case PNS thresholds.

The resulting Impulse head gradient coil underwent testing at the factory and then at Berkeley after integration into the scanner. Performing very high‐resolution EPI in functional MRI (0.6‐mm isotropic resolution and higher), our first development challenge was an unusual mechanical resonance with a five‐pointed star radial pattern occurring in the anterior support structure extension of the gradient coil, which was corrected by integrating a stiffening support ring structure in a replacement gradient coil. Over subsequent months of testing, vibrations from the gradient coil while running high‐resolution EPI sequences loosened connections to external cooling tubes and was remedied with a different connector design. To reduce acoustic noise, we differentiated potential mechanical vibrations from eddy current–induced vibrations localized in the transmit‐receive array coils, specifically in the RF shielding, permitting a 10‐dB reduction in acoustic noise level by modifying the RF shield, while improving the transmit B_1_^+^ efficiency.^10^

The usable performance of the Impulse gradient coil was based on the factory PNS measurements and led to a Stimulation Approximation by Filtering and Evaluation (SAFE) model^18^ implementation on the scanner aimed at predicting PNS and preventing subjects' discomfort. The resulting SAFE model was consistent with the simulated PNS‐threshold predictions by Davids et al.^1^ However, after the gradient coil was integrated into the final scanner setup at Berkeley using different volunteers, PNS was sometimes experienced at lower thresholds on the x‐gradient axis commonly used for EPI axial imaging than anticipated from either the PNS‐optimized model or by the factory PNS measurements that were made with the gradient coil outside of the scanner. The goal of this report was to establish measures of PNS threshold using more subjects, and to investigate origins of discrepancy between the PNS‐optimization prediction model and measured thresholds at two different sites while observing possible effects of subject age, sex, and Z‐position within the gradient coil.

## 2 METHODS

The study at Berkeley was performed on 29 volunteers (18 males, 11 females), age 52.2 ± 17.1 years (average ± standard deviation, age range 30–80 years), and weight 74.0 ± 14.0 kg. Data collection was approved by the Institutional Review Board at UC Berkeley (CPHS no. 2020‐07‐13 437). Written consent was collected from each volunteer, and volunteers were compensated for their participation. The stimulation pulse sequence consisted of a train of 128 alternating bipolar trapezoidal pulses (an EPI readout sequence) with varying rise times to reach a constant gradient amplitude with a 500‐μs plateau duration. This switched‐gradient polarity pulse sequence was used to evaluate each gradient axis separately. The correct gradient amplitudes and rise times were verified with a Skope (Skope MRT, Zürich, Switzerland) field camera.

Volunteers were positioned with their head inside an 8‐transmit, 64‐receive head RF coil (MR CoilTech, Glasgow, UK), placed on the table such that the volunteers' eyebrows (glabella) would be at isocenter when the head was inserted as far as possible into the RF coil, and when the patient table position thus was moved as far as it could (i.e., until 1 mm from contact with the patient bore liner covering the front of the gradient coil). This was done to maximize the overlap with the maximum gradient linearity region. This head position was consistent with the simulations in Davids et al.^1^

We used the method of measuring PNS threshold, as in previous publications.^1^, ^3^, ^13^ The EPI read‐gradient waveform was played with the following rise times: 0.1, 0.22, 0.3, and 0.5 ms (0.2 ms was avoided to skip a mechanical resonance). The zero‐to‐peak gradient amplitude ΔG was then progressively increased in steps of 20 mT/m for each rise time (τ) until volunteers first reported PNS, at the lowest level of sensation. Note that we apply a stimulus of amplitude 2ΔG and duration 2τ when switching from a positive to negative gradient.

Refinements in steps of 2 mT/m were then performed to converge toward the volunteer's specific onset of PNS. When in doubt of PNS, volunteers were instructed to communicate the absence of sensation. The gradient orientation was switched from series to series to cover a single gradient axis (x or y or z) at a time. The whole process was repeated after moving the patient bed, RF coil, and subject 2 cm and 4 cm out toward the feet to identify possible changes in PNS versus Z‐position. Twenty‐nine subjects performed the tests of the x‐gradient axis with the head at isocenter, while 18 of these volunteers did the same for the three gradient axes at the three different Z‐positions. At isocenter, moreover, volunteers were asked to tilt and rotate their heads to likewise determine a possible influence of head orientation inside the coil. The waveform was played out for a few minutes at the subjects' PNS thresholds while the subject moved their head randomly to explore whether tilting could make a difference in PNS. Subjects were instructed to report the location of their sensations whenever they experienced PNS.

We compared the Berkeley measured stimulation limits with those obtained from an earlier PNS testing with the gradient coil out of the scanner at the factory in Erlangen, Germany.^1^ That study was performed under ethics approval with written informed consent on 32 volunteers (16 males, 17 females), age 58.3 ± 13.5 years (average ± standard deviation, age range 21–74 years), and weight 76.6 ± 12.6 kg. A foam headrest (Figure 1D) was positioned on the patient table such that a volunteer's eyebrows (glabella) would be at isocenter when the patient table was moved into the bore up to the bore liner. Volunteers were placed on the patient table with their head on the foam headrest and moved into the bore. The EPI waveform was played in the same fashion as at Berkeley but with the rise times between 0.1 and 0.8 ms in steps of 0.1 ms.

Logistic regression was used for fitting PNS^3^ as a function of zero‐to‐peak rise times (*τ*) and gradient amplitudes (Δ *G*).^2^ The model for the binary output PNS (present or not present), *S*, can be defined as follows:

(1) $$S=\frac{1}{1+e^{-\left(\beta_{0}+\beta_{1}\tau +\beta_{2}\Delta G\right)}}$$

where *β*_*m*_ are the coefficients. To include *N* additional fitting parameters (e.g., age, sex, Z‐position), an additional 2 *N* coefficients can be added to *S* as follows:

(2) $$S=\frac{1}{1+e^{-\left(\beta_{0}+\beta_{1}\tau +\beta_{2}\Delta G+\sum_{n=1}^{N}\left(\beta_{1+2n}{\phi^{\prime }}_{n}+\beta_{2+2n}{\phi^{\prime }}_{n}\;\tau \right)\right)}}$$

where ${\phi^{\prime }}_{n}=\phi_{n}-\bar{\phi_{n}}$; and $\bar{\phi_{n}}$ is the population mean of parameter *n*, except for Z‐position where $\bar{\phi_{n}}$ is set to 0. The coefficients from the logistic regression model using different sets of parameters can be used to predict PNS thresholds^2^, ^3^ for a given combination of parameters (e.g., rise time, age, sex, Z‐position) via $\Delta G={\Delta G}_{\min}+\tau \cdot SR_{\min}$, using

(3) $$\begin{array}{cc} {\Delta G}_{\min} & =-\frac{\beta_{0}}{\beta_{2}}-\sum_{n=1}^{N}\frac{\beta_{1+2n}}{\beta_{2}}\;{\phi^{\prime }}_{n}\;\text{and}\;SR_{\min}\\ & =-\frac{\beta_{1}}{\beta_{2}}-\sum_{n=1}^{N}\frac{\beta_{2+2n}}{\beta_{2}}\;{\phi^{\prime }}_{n}. \end{array}$$

We implemented the logistic regression fitting using the generalized linear regression model library of *MATLAB* (MathWorks, Natick, MA, USA) and used the fraction of volunteers that stimulated at each rise time and axis as fitting weights.

PNS threshold calculation was also performed after combining the UC Berkeley and factory data. Logistic regression was performed twice: once including age and sex as additional parameters (for the UC Berkeley, factory, and combined datasets) and one including age, sex, and Z‐position (UC Berkeley only).

Additional to the experimental work, we used the PNS modeling workflow to assess the effect of the subject's position within the coil on the expected PNS thresholds. We varied the position of both the male and female body models in 5‐mm steps by up to ±40 mm along x‐ and y‐directions and by ±50 mm along the Z‐direction and assessed the predicted variations in PNS thresholds at 300‐us rise time. The body‐model shifts were constrained to avoid intersections between the body model and the bore liner.

For each testing position at UC Berkeley, we shimmed the three‐dimensional brain and performed localizer sagittal scans (two‐dimensional (2D) gradient echo (GRE), 0.5‐mm in‐plane resolution, 3‐mm thickness, bandwidth = 1950 Hz/pixel) to control head position with respect to isocenter. Additional 2D‐GRE acquisitions (0.22‐mm in‐plane resolution, 1‐mm thickness) were also performed to inspect the nonlinearity distortions associated with the shifts. Images were reconstructed with and without distortion correction for gradient nonlinearities.

Simulations were performed to demonstrate the different combinations of gradient amplitude and rise time to achieve a particular echo spacing^19^ for three different resolutions, to investigate the potential impacts of lowered PNS thresholds on EPI‐based neuroimaging. The minimum required gradient amplitude and rise time for any given echo spacing is the local minimum (right corner point) in each iso‐echo spacing curve, above which a higher gradient amplitude has no effect on reducing echo spacing.

## 3 RESULTS

Tables 1 and 2 show data from measurements at isocenter at the factory and at Berkeley. Tables S1 and S2 show data from measurements at Berkeley at 2 and 4 cm offset, respectively. The data are provided to enable other analyses and comparisons with other gradient coils, beyond the scope of this report.

**TABLE 1:** Peripheral nerve stimulation (PNS) thresholds (zero‐to‐peak, in mT/m) for individual subjects from the Berkeley data, per rise time per gradient axis, up to system hardware limits. Subjects who experienced no PNS for that gradient axis and rise time are marked “No PNS.” Subjects who were not tested on that gradient axis are marked with a “–.”

Berkeley																			
					GX	Rise times (us)				GY	Rise times (us)				GZ	Rise times (us)			
Subject	Sex	Age (year)	Height (cm)	Weight (kg)	Location	100	220	300	500	Location	100	220	300	500	Location	100	220	300	500
1	M	34	172	73.5	Forehead/Nose	62	82	96	116	Shoulder	90	106	132	136	Upper chest	74	72	88	122
2	M	46	177	80	Forehead	52	74	92	124	Chest/Arms	76	112	128	166	Upper Chest	64	96	108	128
3	F	32	175	68	Forehead/Nose	74	84	102	130	Arms	92	118	146	196	Chest	68	108	136	180
4	F	33	160	63	Nose	64	82	88	120	Arms	84	124	150	196	Upper chest	82	108	122	No PNS
5	M	38	178	79	Nose	74	100	116	138	Nose/Shoulders	78	114	134	178	Chest/Shoulders	66	88	106	140
6	F	39	172	100	Nose	48	64	70	84	Shoulders	84	124	150	No PNS	Chest/Shoulders	68	92	106	132
7	M	39	172	70	Nose	74	122	148	No PNS	Shoulders	No PNS	142	166	No PNS	Shoulders	80	102	124	182
8	F	42	172	72	Nose	No PNS	128	144	164	–	–	–	–	–	Shoulders	64	84	98	126
9	F	55	165	70	Forehead	80	106	110	172	–	–	–	–	–	Chest	76	102	114	142
10	M	75	185.4	74.8	Nose/Forehead	No PNS	160	172	178	Shoulders	28	120	142	No PNS	Shoulders	72	116	142	No PNS
11	F	40	170.18	86.18	Nose	64	80	90	102	Neck/Shoulders	No PNS	120	160	No PNS	Armpit	56	78	92	140
12	M	37	185	84	Forehead	No PNS	124	122	166	Shoulders/Hands	No PNS	124	144	No PNS	Chest	80	104	122	156
13	M	68	169	75	N/A	No PNS	No PNS	No PNS	No PNS	N/A	No PNS	No PNS	No PNS	No PNS	Chest	No PNS	No PNS	No PNS	No PNS
14	M	68	180.3	97		No PNS	No PNS	No PNS	No PNS	Shoulders	No PNS	176	196	No PNS	Chest	76	118	136	198
15	F	72	157.48	52.6	Forehead/Nose	86	120	144	182	Hands	86	122	146	No PNS	Chest	80	102	126	144
16	M	30	184	89		46	62	90	120		72	92	112	164		56	70	80	98
17	M	41	183	83	Nose	52	80	96	140	Shoulders	82	100	126	180	Chest	72	124	130	172
18	M	48	193	95	Eyebrow	64	90	112	138	Shoulders	88	124	152	198	Shoulders	80	110	120	158
19	M	32	160	68	Nose	No PNS	108	134	160	Lower back	78	110	130	156	Shoulders	58	78	88	90
20	F	72	178	81.65	N/A	No PNS	No PNS	No PNS	No PNS	–	–	–	–	–	–	–	–	–	–
21	F	70	152	52.2	Eyebrow	68	116	136	No PNS	–	–	–	–	–	–	–	–	–	–
22	M	73	178	74.8	Forehead/Nose	60	60	82	108	–	–	–	–	–	–	–	–	–	–
23	M	80	177	74.84	Nose	No PNS	152	172	No PNS	–	–	–	–	–	–	–	–	–	–
24	F	72	157	51.26	Nose	No PNS	170	198	No PNS	–	–	–	–	–	–	–	–	–	–
25	M	33	160	54	Nose	74	92	110	152	–	–	–	–	–	–	–	–	–	–
26	M	68			Nose	60	98	130	194	Shoulder	No PNS	110	126	186	Shoulder	No PNS	118	114	164
27	F	43	165	54.43	Nose	68	102	120	166	Shoulder	No PNS	114	176	No PNS	Shoulder	60	80	78	92
28	M	60	172	90.71	N/A	No PNS	No PNS	No PNS	No PNS	Chest	No PNS	132	No PNS	No PNS	Chest	No PNS	112	No PNS	No PNS
29	M	74	176	58	Head	78	164	No PNS	No PNS	Head	No PNS	172	No PNS	No PNS	Shoulders	80	146	172	No PNS

**TABLE 2:** Peripheral nerve stimulation (PNS) thresholds (zero‐to‐peak, in mT/m) for individual subjects from the Erlangen data, per rise time per gradient axis. Subjects who experienced no PNS for that gradient axis and rise time are marked “No PNS.” PNS location for that axis is listed if recorded.

Erlangen																															
					GX	Rise times (us)								GY	Rise times (us)								GZ	Rise times (us)							
Subject	Gender	Age (years)	Height (cm)	Weight (kg)	Location	100	200	300	400	500	600	700	800	Location	100	200	300	400	500	600	700	800	Location	100	200	300	400	500	600	700	800
1	M	74	172	73	N/A	No PNS	No PNS	No PNS	No PNS	No PNS	No PNS	No PNS	No PNS	N/A	No PNS	No PNS	No PNS	No PNS	No PNS	No PNS	No PNS	No PNS	Chest	No PNS	No PNS	200	No PNS	No PNS	No PNS	No PNS	No PNS
2	M	68	178	93	Forehead	65	93	123	155	180	No PNS	No PNS	No PNS	Lower jaw	No PNS	128	142	153	163	192	No PNS	No PNS	Scapula	80	98	107	128	145	169	190	200
3	F	57	168	95	N/A	No PNS	No PNS	No PNS	No PNS	No PNS	No PNS	No PNS	No PNS	Shoulder Forearm	No PNS	181	200	No PNS	No PNS	No PNS	No PNS	No PNS	Upper arm	No PNS	130	150	170	200	No PNS	No PNS	No PNS
4	M	57	187	93	N/A	No PNS	No PNS	No PNS	No PNS	No PNS	No PNS	No PNS	No PNS	Temple/Neck	No PNS	160	190	No PNS	No PNS	No PNS	No PNS	No PNS	Neck	90	130	160	180	No PNS	No PNS	No PNS	No PNS
5	M	67	172	93	Forehead Nose	No PNS	140	165	No PNS	No PNS	No PNS	No PNS	No PNS	Forehead/Upper Arm	No PNS	145	195	No PNS	No PNS	No PNS	No PNS	No PNS	Chest	No PNS	135	165	190	No PNS	No PNS	No PNS	No PNS
6	F	64	164	68	Upper jaw	No PNS	160	170	No PNS	No PNS	No PNS	No PNS	No PNS	Shoulder	85	110	120	125	155	165	175	190	Scapula	85	105	130	150	185	No PNS	No PNS	No PNS
7	M	67	180	90	Forehead	91	112	136	188	No PNS	No PNS	No PNS	No PNS	Shoulder	No PNS	148	172	195	No PNS	No PNS	No PNS	No PNS	Shoulder	No PNS	133	156	No PNS	No PNS	No PNS	No PNS	No PNS
8	M	51	183	72	Nose	87	101	114	127	147	180	No PNS	No PNS	Forehead	91	140	168	193	No PNS	No PNS	No PNS	No PNS	Shoulder	No PNS	140	174	No PNS	No PNS	No PNS	No PNS	No PNS
9	F	52	164	74	N/A	No PNS	No PNS	No PNS	No PNS	No PNS	No PNS	No PNS	No PNS	Shoulder	85	112	135	160	188	No PNS	No PNS	No PNS	Scapula	89	110	129	136	158	171	190	No PNS
10	M	61	186	90	N/A	No PNS	No PNS	No PNS	No PNS	No PNS	No PNS	No PNS	No PNS	Shoulder/Scapula	No PNS	150	185	No PNS	No PNS	No PNS	No PNS	No PNS	Shoulder Armpit	No PNS	120	155	190	200	No PNS	No PNS	No PNS
11	F	71	170	64		No PNS	178	193	No PNS	No PNS	No PNS	No PNS	No PNS		No PNS	151	181	No PNS	No PNS	No PNS	No PNS	No PNS		No PNS	162	177	No PNS	No PNS	No PNS	No PNS	No PNS
12	F	47	168	52	N/A	No PNS	No PNS	No PNS	No PNS	No PNS	No PNS	No PNS	No PNS	N/A	No PNS	No PNS	No PNS	No PNS	No PNS	No PNS	No PNS	No PNS		No PNS	145	No PNS	No PNS	No PNS	No PNS	No PNS	No PNS
13	F	21	154	54	N/A	No PNS	No PNS	No PNS	No PNS	No PNS	No PNS	No PNS	No PNS	Shoulder	No PNS	140	No PNS	No PNS	No PNS	No PNS	No PNS	No PNS	Shoulder	No PNS	130	150	No PNS	No PNS	No PNS	No PNS	No PNS
14	M	31	188	87	Forehead	No PNS	175	No PNS	No PNS	No PNS	No PNS	No PNS	No PNS	Neck	86	114	129	147	163	185	198	No PNS	Shoulder/Neck	73	91	103	120	136	148	153	179
15	M	58	183	92	Nose	80	105	120	140	155	175	190	No PNS	Shoulder	85	110	120	145	155	175	190	No PNS	Scapula	75	95	105	120	130	140	150	160
16	F	52	175	85	Forehead	No PNS	160	170	No PNS	No PNS	No PNS	No PNS	No PNS	Shoulder	No PNS	116	128	135	147	165	173	191	Shoulder	No PNS	124	132	160	177	198	No PNS	No PNS
17	F	67	157	63	N/A	No PNS	No PNS	No PNS	No PNS	No PNS	No PNS	No PNS	No PNS	Shoulder/Hand	No PNS	165	200	No PNS	No PNS	No PNS	No PNS	No PNS	Chest Hand	No PNS	124	133	178	No PNS	No PNS	No PNS	No PNS
18	F	73	169	68	N/A	No PNS	No PNS	No PNS	No PNS	No PNS	No PNS	No PNS	No PNS	Hand	No PNS	170	190	No PNS	No PNS	No PNS	No PNS	No PNS	Lip	No PNS	160	No PNS	No PNS	No PNS	No PNS	No PNS	No PNS
19	F	24	168	65	Nose	No PNS	152	166	No PNS	No PNS	No PNS	No PNS	No PNS	Neck	91	108	132	156	176	199	No PNS	No PNS	Chest	91	109	124	137	156	173	200	No PNS
20	F	48	168	69	Nose	90	120	130	150	160	190	195	No PNS	Scapula/Neck	No PNS	135	155	180	No PNS	No PNS	No PNS	No PNS	Scapula	73	103	118	145	150	160	165	170
21	F	62	160	65	N/A	No PNS	No PNS	No PNS	No PNS	No PNS	No PNS	No PNS	No PNS	N/A	No PNS	No PNS	No PNS	No PNS	No PNS	No PNS	No PNS	No PNS	N/A	No PNS	No PNS	No PNS	No PNS	No PNS	No PNS	No PNS	No PNS
22	F	51	170	82	Nose	72	83	99	114	127	140	158	175	Shoulder	75	85	92	109	120	129	134	151	Neck Chest	89	104	122	151	177	184	194	No PNS
23	M	65	180	80	Nose	No PNS	162	173	196	No PNS	No PNS	No PNS	No PNS	Scapula	No PNS	146	174	191	No PNS	No PNS	No PNS	No PNS	Scapula	No PNS	125	138	150	172	195	No PNS	No PNS
24	F	53	169	70	Nose	90	120	140	150	180	190	No PNS	No PNS	Scapula	90	110	140	160	180	200	No PNS	No PNS	Chest	80	120	130	130	140	150	150	180
25	M	68	186	82	N/A	No PNS	No PNS	No PNS	No PNS	No PNS	No PNS	No PNS	No PNS	Scapula	No PNS	137	155	173	192	No PNS	No PNS	No PNS	Shoulder	74	101	120	134	159	174	200	No PNS
26	F	70	168	74	N/A	No PNS	No PNS	No PNS	No PNS	No PNS	No PNS	No PNS	No PNS	N/A	No PNS	No PNS	No PNS	No PNS	No PNS	No PNS	No PNS	No PNS	N/A	No PNS	No PNS	No PNS	No PNS	No PNS	No PNS	No PNS	No PNS
27	M	73	191	83	Nose	No PNS	170	180	No PNS	No PNS	No PNS	No PNS	No PNS	Collarbone	No PNS	130	170	190	No PNS	No PNS	No PNS	No PNS	Chest	90	130	150	170	190	No PNS	No PNS	No PNS
28	F	59	162	87	Nose	No PNS	73	190	No PNS	No PNS	No PNS	No PNS	No PNS	Shoulder/Armpit	No PNS	137	143	168	171	No PNS	No PNS	No PNS	Chest	63	80	83	101	114	122	128	152
29	M	73	174	73	Upper arm	No PNS	170	190	No PNS	No PNS	No PNS	No PNS	No PNS	Collarbone	No PNS	140	180	No PNS	No PNS	No PNS	No PNS	No PNS	Chest	90	120	150	170	190	No PNS	No PNS	No PNS
30	M	67	183	89	Neck	No PNS	195	No PNS	No PNS	No PNS	No PNS	No PNS	No PNS	Ear/Scapula	No PNS	172	No PNS	No PNS	No PNS	No PNS	No PNS	No PNS	Shoulder	No PNS	140	164	192	No PNS	No PNS	No PNS	No PNS
31	M	65	183	73	Forehead	No PNS	180	No PNS	No PNS	No PNS	No PNS	No PNS	No PNS	Neck	90	120	140	160	180	200	No PNS	No PNS	Shoulder Scapula	60	110	120	130	140	150	160	170
32	F	49	167	53	N/A	No PNS	No PNS	No PNS	No PNS	No PNS	No PNS	No PNS	No PNS	Scapula	87	156	No PNS	No PNS	No PNS	No PNS	No PNS	No PNS	Scapula	No PNS	127	152	187	No PNS	No PNS	No PNS	No PNS

**FIGURE 2:**
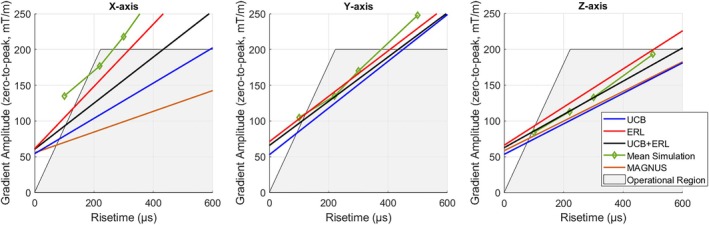
Comparison of measured thresholds at UC Berkeley and factory. Logistic fit measurements from Berkeley at isocenter (UCB; *blue*) and in the factory at Erlangen (ERL; *red*). Peripheral nerve stimulation (PNS) modeling results (*green*) redrawn from Davids et al.^1^ showing simulated average values are included for reference. Logistic fit measurements using the combined data from Berkeley and Erlangen (UCB + ERL) are shown in black. The x‐ and z‐axes PNS thresholds for a conventional shielded two‐layer head‐only gradient as reported by Tan et al.^2^ (MAGNUS, Gmax = 200 mT/m, Shinnar le Roux [SLR] = 500 mT/m/ms) are shown for comparison. The y‐axis thresholds were not calculated in Tan et al.^2^ due to insufficient subjects experiencing stimulation on this axis. The gradient operational region of Impulse gradient (*gray*) is defined by SLR = 900 mT/m and Gmax = 200 mT/m/ms. The β‐parameters of the logistic regression model are provided in Table S3.

Figure 2 shows a comparison of Berkeley isocenter and factory measurements^1^ relative to modeled PNS thresholds for each gradient axis. Although good agreement between the results can be observed for the y‐ and z‐axes, lower PNS thresholds were characterized in Berkeley for the x‐axis. Interestingly, no major differences were found in either the Berkeley or factory measurements for males and females (see Table S3), in line with previous studies^2^ but contrary to the simulated curves from the modeling,^1^ which used two different size cadavers for male and female modeling (male height: 176 cm, weight: 81.6 kg; female height: 162.6 cm, weight: 52.6 kg). The logistic fit using the combined data from Berkeley and the factory is also shown.

For the x‐axis, volunteers primarily reported the sensations to be located on the face between the eyes, on the nose, and/or the forehead. Because of those locations, volunteers affirmatively confirmed that the PNS could not be misinterpreted as vibrations. For the y‐ and z‐axes, locations of the PNS were in the shoulders, chest, and hands.

**FIGURE 3:**
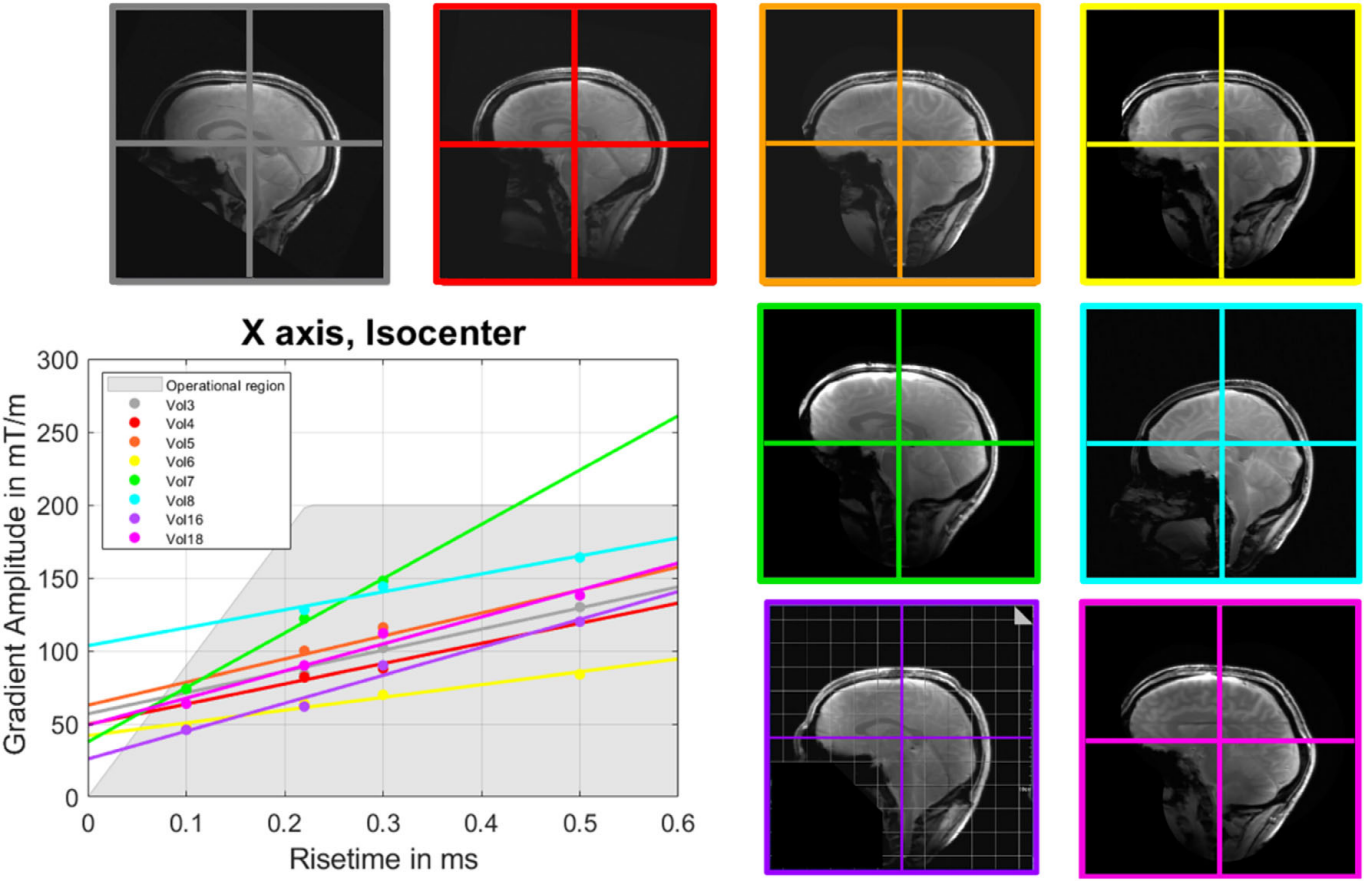
Localizer images with corresponding peripheral nerve stimulation data. Head localizer images for various subjects demonstrate consistency of isocenter positioning (*crosshairs*) across subjects despite variability of head sizes and shapes. Corresponding colors on the plot demonstrate variability of threshold curves despite similar positioning at isocenter among subjects.

In Figure 3, brain localizer images acquired at UC Berkeley for various subjects demonstrate consistency of isocenter positioning across subjects. The average position of the glabella relative to isocenter when the patient bed was moved in as far as possible, as measured from the localizers, was −0.4 ± 10.67 mm (average ± SD), indicating consistent placement of this anatomical landmark at isocenter. Nevertheless, one can appreciate that the spread of measured threshold curves for the x‐axis for each individual across rise times was relatively wide. Individual PNS thresholds and linear fits of stimulation level across all 29 volunteers for the x‐gradient axis at isocenter are shown in Figure S3. Tilting the head within the receiver coil at isocenter did not have a significant effect on the PNS thresholds reported by 4 volunteers, ruling out this factor as an explanation for the discrepancy between the experimental results obtained at Berkeley and the factory.

**FIGURE 4:**
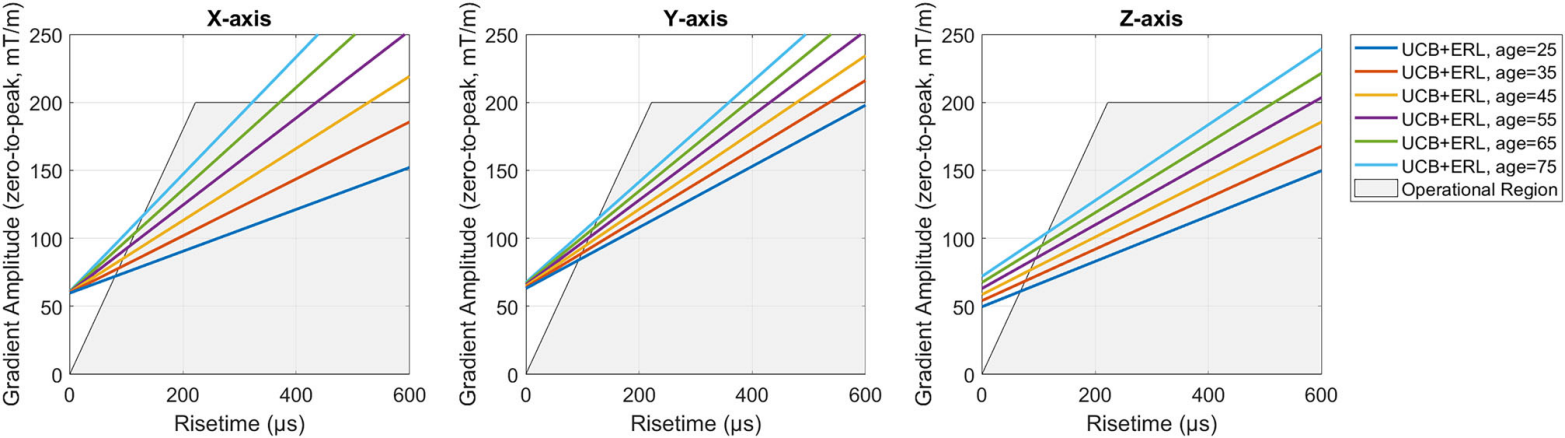
Effect of age on peripheral nerve stimulation thresholds. Individual plots of threshold by age can be found in Figures S3 and S4. ERL, Erlangen; UCB, UC Berkeley.

Figure 4 shows the predicted effects of age on PNS thresholds calculated from the combined data collected at isocenter at UC Berkeley and the factory.

**FIGURE 5:**
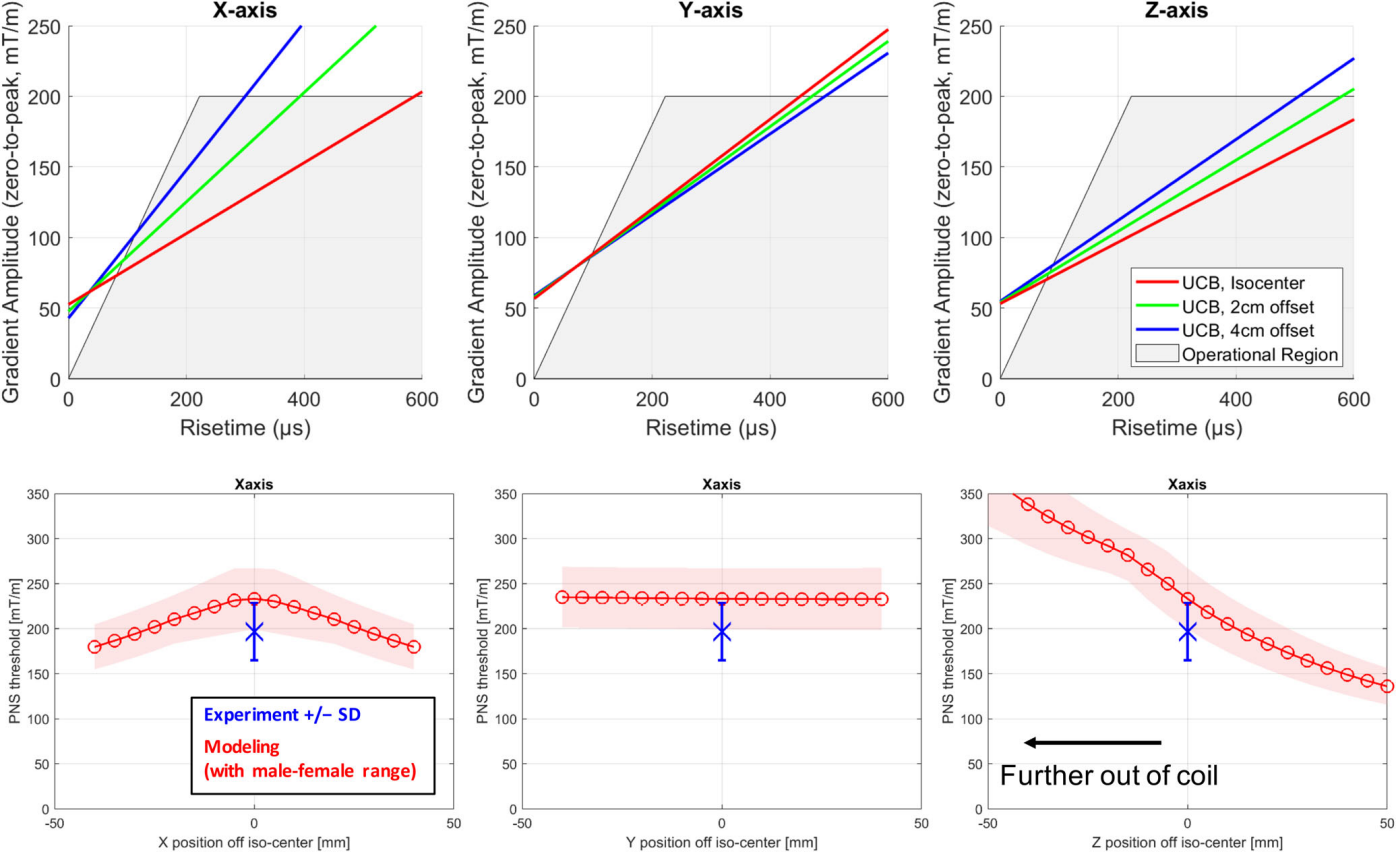
Experimental and simulated peripheral nerve stimulation (PNS) thresholds versus positioning. *Top row*: Measurements of PNS threshold performed with NexGen 7T scanner at Berkeley at 0‐cm isocenter, 2 cm from isocenter in the foot direction, and 4 cm from isocenter. *Bottom row*: Modeling results showing x‐axis thresholds with three directions of displacement at 300‐μs rise time, plotted alongside the experimental data from the factory at Erlangen. Simulations for y‐axis and z‐axis are shown in Figure S1. The β‐parameters of the logistic regression model are provided in Table S4. SD, standard deviation; UCB, UC Berkeley.

Figure 5 (top row) displays the predicted stimulation thresholds at isocenter 0, 2 cm from isocenter, and 4 cm from isocenter in the head–foot direction from logistic regression of the data from UC Berkeley for the three gradient axes, revealing a dependence of PNS on z‐position mostly for the gradient x‐axis. Figure 5 (bottom row) shows the x‐offset, y‐offset and z‐offset sensitivities for the Impulse x‐axis, modeled at 300‐μs rise time, plotted alongside the experimental data from the factory at Erlangen. The z‐position dependence matches the trends seen in the experimental findings. Note the predicted reduced threshold in the right half of the model X curve by head displacements into the magnet beyond isocenter is prevented by the shoulders being up against the smaller diameter of the gradient coil. The effect of position for the y‐ and z‐axes can be seen in Figure S1, although these shifts could not be done experimentally due to the physical restrictions of the gradient coil.

**FIGURE 6:**
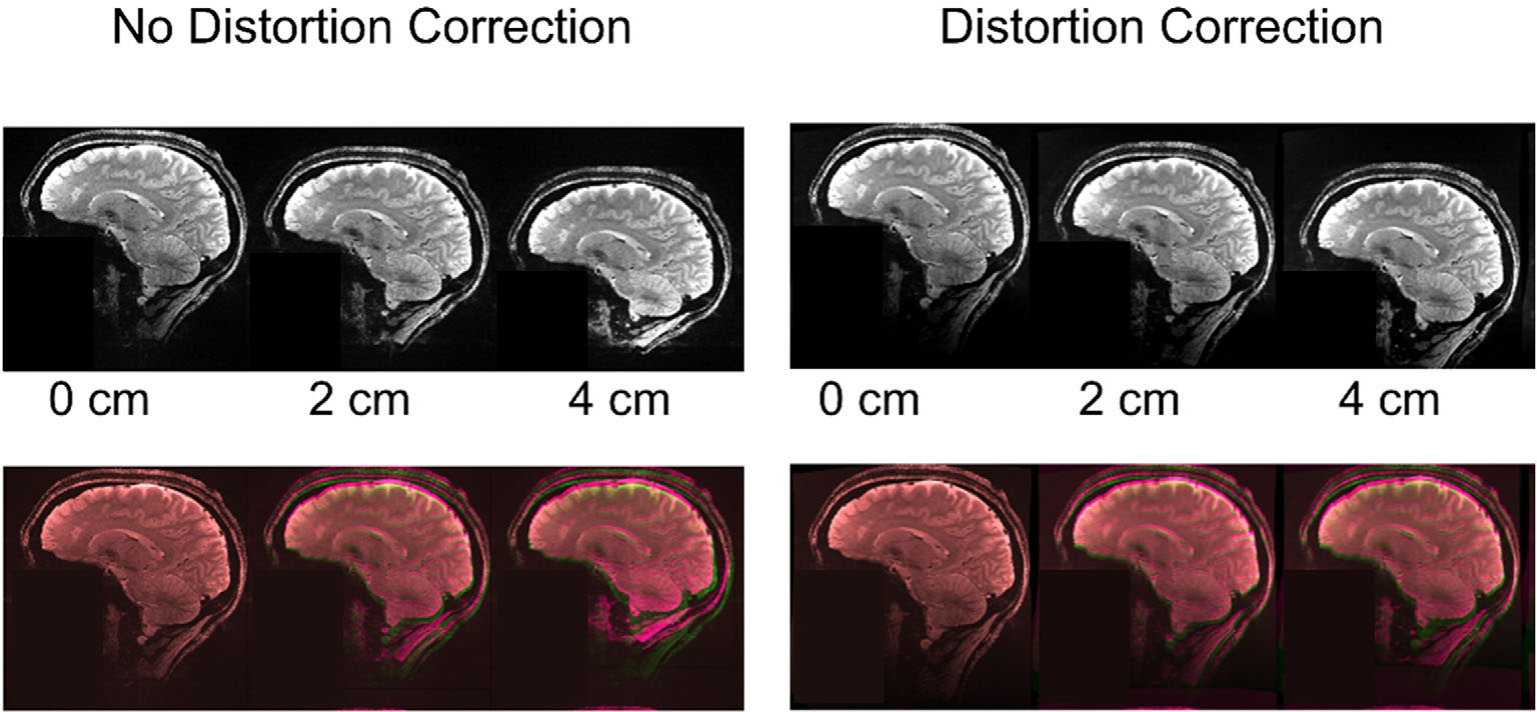
Nonlinearity distortions with 220‐μm in‐plane resolution two‐dimensional gradient‐echo image. *Top*: The different images acquired at isocenter (0 cm), 2 cm, and 4 cm toward the feet are shown without (*left*) and with (*right*) distortion corrections. Bottom: Same as top but with the images translated by the respective displacements and superposed to the one located at isocenter to illustrate the blurring.

Figure 6 shows 220‐μm in‐plane‐resolution 2D‐GRE sagittal images of the head, revealing distortion at the different Z‐positions. After applying the nonlinearity correction algorithms, the heads recovered their original overall shapes to a large extent, yet with remaining imperfections, leading to resolution loss in lower parts of the brain. The same illustrations for a spherical agar gel phantom of 15.6‐cm diameter can be found in Figure S2.

**FIGURE 7:**
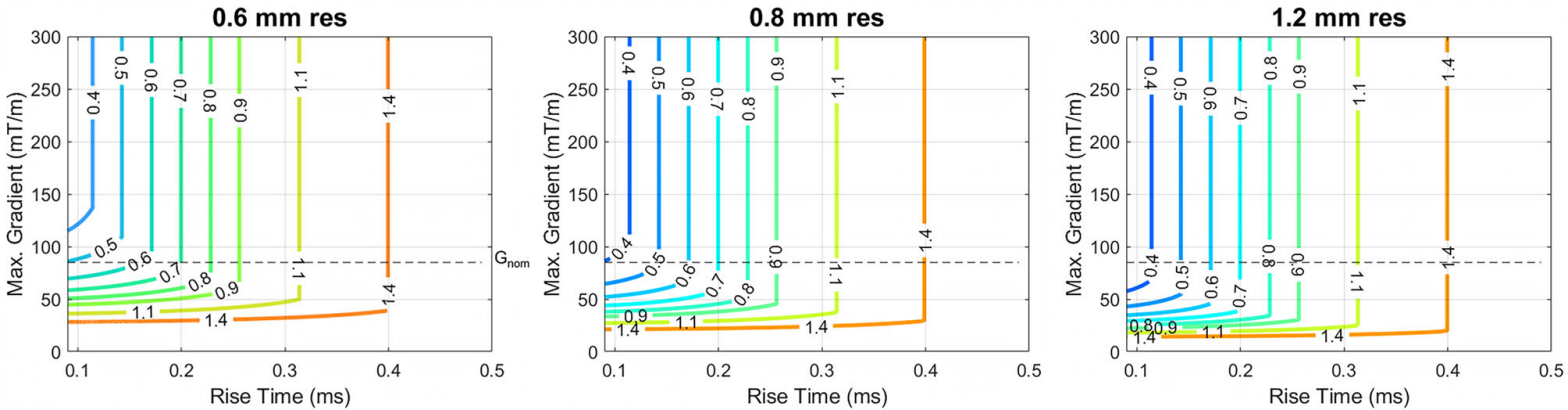
Contour lines of echo spacing (ms) versus gradient amplitude (mT/m) and gradient rise time (ms) for 0.6‐, 0.8‐, and 1.2‐mm isotropic resolution, respectively, assuming no ramp sampling. The value of G_nom_ is 85 mT/m.

Figure 7 shows the contour lines of echo spacing versus gradient amplitude and rise time for 0.6‐, 0.8‐, and 1.2‐mm isotropic resolution in EPI, assuming no ramp sampling.

## 4 DISCUSSION

We have carefully evaluated minimum PNS stimulation thresholds and determined the x‐gradient axis to be considerably lower than previously reported in experimental and theoretical work (Figure 2). At the factory, over half the subjects' PNS onset thresholds could not be reached for the x‐gradient axis,^1^ even at the highest gradient amplitudes allowed by the hardware. The discrepancy between the experimental measurements made at Berkeley and at the factory could be explained by variability in subject anatomy, and/or differences in the age distributions of the cohorts, and/or different positions of the subjects in the gradient coil, discussed subsequently. Although we have demonstrated the effect of these variables on PNS thresholds, the relative weighting of these or other factors in explaining the discrepancy between the two sites, and with the PNS modeling, has not been shown. Note the dependence of PNS threshold on position has also been seen in previous studies^2^ and was confirmed with additional simulations (Figure 5B).

Figure 4 shows the effects of age on PNS thresholds using the combined data sets from UC Berkeley and the factory. PNS thresholds increase with age on all three gradient axes, with greater increase for the x‐axis. The cohort in the factory data^1^ had a mean age of 58.3 years with only 3 volunteers under 45 years old, which could have resulted in an upward shift of the curve in the previously published data.^1^, ^13^ There was potentially other physical variability in cohorts (i.e., head and body circumferences), which might correlate with differences in PNS thresholds, that were not evaluated due to the small sample size.

The overall sensitivity of PNS thresholds to the subject's Z‐position is important to note, as at the time of the earlier experiment at the factory, a full scanner system was not available. There was also no RF coil available at the factory; thus, subjects' heads were held in a concave foam cushion at isocenter to approximate RF coil positioning. Subject positioning was constrained by positioning of the foam head rest; however, without imaging capabilities, the head position relative to isocenter could not be verified and could potentially be changed by subjects, which may have increased the PNS threshold.

As shown in Figure 5A, the PNS threshold increased by about 20 mT/m per z‐axis centimeter displacement toward the feet, in good agreement with the simulations (Figure 5B) and in line with previous studies.^2^ Our results show that asymmetric head gradients exhibit stronger Z‐offset‐based PNS threshold dependence than do symmetric gradient coils. The new simulation (Figure 5B) could nicely reproduce the sensitivity of the PNS thresholds of the x‐gradient versus the z‐position.

Although the discrepancy between the new and prior experimental data could be explained by different age distribution, position inconsistencies, or subject cohort variations, the PNS model in Davids et al.^1^ predicted a much higher threshold on the x‐axis than either the Berkeley data alone, or the combined Berkeley and factory experimental results (Figure 2). Although the PNS model was used primarily to balance stimulation thresholds between head and shoulders, the x‐gradient in our experiments only stimulated the head. If only the factory cohort is considered, this would suggest there was some level of PNS improvement arising from the model informed design. Agreement with the model for the other two gradient axes was within the predictions. It should be noted that although the x‐axis PNS thresholds demonstrated on the UC Berkeley scanner are lower than those from the factory, the x‐axis thresholds of this novel three‐layer coil are still higher than conventional shielded two‐layer head gradient coils,^20^ such as the GE MAGNUS (Figure 2). PNS thresholds for z‐axis look more comparable with MAGNUS (as expected, as the Impulse z‐gradient is symmetric and not optimized), and PNS thresholds for the y‐axis are not comparable, as they could not be calculated for MAGNUS gradient.^2^ Furthermore, the Impulse gradient has a higher linearity than MAGNUS (11% compared with 18%, respectively, calculated for 26‐cm field of view^1^), allowing good centric head positioning, and has a large 44‐cm inner diameter for RF coils and visual fields.

The model also reported stimulation thresholds of the female x‐axis curve as significantly higher than stimulation thresholds of males, which in this and previous work^2^ could not be reproduced experimentally. The reason for this difference in predicted PNS thresholds may be due to the different head sizes of the two models, as shown in Davids et al.,^11^ and slight anatomical differences between the two models; however, whatever the causal factors are, they are not known for the modeling methodology. There is similarity in facial nerve location and diameter between sexes,^21^ so the difference in the model simulation between male and female presumably was primarily due to body size being greater in the male model. Furthermore, our experimental results did not show significant differences between males and females even for the y‐ and z‐axes, where PNS mostly occurred in shoulders, hands, and chest. This finding is consistent with previous work,^2^ and given the variability of PNS thresholds experienced by the volunteers, the reported experimental data now suggest that the spread among the two models in the simulations is more related to variability in body size, as opposed to sex. Additionally, the fine anatomical structures in the head (especially the thin layer of conductive tissue in the face) render electric‐field simulations complicated, and the resulting electric fields are more strongly affected by the fine anatomical features, such as small bundles of facial muscles.

We are in the process of identifying potential reasons for the disagreement between model‐predicted and experimental thresholds. One potential source of error could be an incorrect assignment of nerve axon diameters to the facial nerve. Although variations in facial nerve axon diameters between 4 and 6 μm have been reported in the literature,^22^ this level of variability is not sufficient to explain the observed discrepancy. Additional sources of error could be related to the anatomical model (distribution of conductive tissues). Given the reported PNS data, one must acknowledge the large variability of PNS thresholds across subjects in the Impulse head gradient coil, so that the male and female body models may not also likely be representative of the mean of the population, unless further measures are taken to achieve this goal. The anatomical cadaver data used in the PNS neurodynamic models may not have been representative of live subjects either, or refinements for this configuration or for facial nerves are still needed. Recently, such modeling was shown theoretically and experimentally to be successful for whole‐body gradient coils^23^ where stimulations occur more in the body, suggesting again that potentially subtle differences in facial nerves^24^ are giving erroneous model results in the Impulse head gradient coil. Yet, the simulated thresholds match some of the experimental measurements we report for some subjects. Without comparing the experimental PNS data of a non‐optimized head gradient coil of otherwise identical specifications, one cannot firmly conclude on the effectiveness of the simulations and the balancing procedure to raise the PNS thresholds. However, one is led to observe larger thresholds on the x‐gradient arising from our combined data than for other head gradient coils, such as MAGNUS,^2^ with more conventional two‐layer designs (Figure 2). Other differing factors such as linearity, inner diameter, and inductance also play a role in the results and do not allow making an accurate and direct comparison.^3^ Although the source of the discrepancies remains to be elucidated, the accuracy of the neurodynamic model for the simulation of the Impulse head gradient coil remains to be demonstrated more convincingly, particularly for the facial region stimulated by the x‐gradient.

The results of the PNS sensitivity versus Z‐position tests indicate that the lower PNS thresholds on the x‐axis could be mitigated if critical, for example, to reduce echo spacing in EPI, by moving the patient table and subject a few centimeters toward the feet and away from isocenter to raise thresholds and recover performance. This approach, however, is limited by greater nonlinearity distortions in the acquired images for displacements larger than 2 cm (Figure 6), and algorithms to correct distortions have a loss of resolution proportional to the distortion.

Another approach to maximizing gradient performance by avoiding PNS, making use of the large spread of PNS thresholds between individuals and age distribution, is to perform subject‐specific determination of PNS threshold using prescan testing with incremented gradient amplitudes to reach a minimum threshold, as performed in our experiments rather than applying a mean threshold limit on all subjects. This could easily be integrated into the protocol with sequence tune‐up and supervision when a new subject is registered, requiring a few minutes of time. Nevertheless, the International Electrotechnical Commission guidelines currently allow 50% of the population to potentially experience PNS beyond the threshold limit based on the mean of the test population, and this would be the case given the spread we observed in our experimental data. Similarly, many individuals may feel increased discomfort for the facial PNS (x‐axis) even with non–echo‐planar imaging.

Usable gradient performance may be limited in several ways. For EPI, usually either the thermal limits of components or PNS restrictions kick in first. At the Berkeley system, the maximum usable gradient amplitude is limited to protect the gradient against overheating during long scans. For EPI in functional imaging, the nominal gradient limit (Gnom) of 85 mT/m^13^ is the maximum usable gradient amplitude to protect the gradient power amplifiers. As shown in Figure 7 using high‐spatial‐resolution EPI of 0.6‐mm isotropic resolution, PNS thresholds within the gradient operating curve would potentially limit the gradient performance below Gnom and increase the minimum echo spacing from 0.58 ms^13^ to longer values. Hence, the lower PNS thresholds may affect optimization of performance for very high‐resolution EPI sequences. For lower‐resolution imaging, shorter echo spacing can be used with no performance limitations from PNS thresholds. Non‐Cartesian (spiral) trajectories may become limited by PNS in achieving higher spatiotemporal resolution.

## 5 CONCLUSION

We characterized PNS threshold dependence on subject age and with respect to Z‐positioning, corroborated by simulations, all validating previous studies.^2^ The discrepancy between the experimental results in the scanner at Berkeley and those acquired at the factory could be attributed to the individual effects or combined effects of the stimulation variations of the subjects, possible variations of subject Z‐positions, and age distribution.

In summary, PNS measurements showed lower PNS thresholds on the x‐axis than predicted by the PNS optimization model or the factory cohort. This discrepancy in the model prediction used to guide the intermediate layer winding pattern remains to be elucidated. There were to a lesser extent lower thresholds on the y‐ and z‐axes compared with model predictions. We believe it is important to report on the new findings of PNS thresholds to avoid unnecessary subject discomfort, as well as to demonstrate variables affecting PNS testing of gradient coils and offer the opportunity to improve gradient‐coil modeling of PNS. Notwithstanding, the Impulse gradient, with its high slew rate and high maximum gradient amplitude, implemented in a 7T scanner with signal‐to‐noise ratio and blood oxygen level–dependent contrast advantages, provides exceptionally high performance for human brain imaging.

## CONFLICT OF INTEREST

D.F., E.W., N.B., and A.B. are employees of Advanced MRI Technologies. S.M. is an employee of Siemens Medical Solutions USA, Inc. D.R., E.R., and P.D. are employees of Siemens Healthcare GmbH.

## Supporting information


**Figure S1.** The y‐axis and z‐axis simulated thresholds with three directions of displacement from modeling.
**Figure S2.** Nonlinearity distortions with 15.6‐cm‐diameter agar spherical phantom. Top: The different localizer sagittal images (0.5 mm in‐plane resolution, 3‐mm thickness) acquired at isocenter (0 cm), 2, 4 and 6 cm toward feet are shown without (*left*) and with (*right*) distortion corrections. Bottom: Same as top but with the images translated by the respective displacements and superposed to the one located at isocenter.
**Figure S3.** Individual peripheral nerve stimulation (PNS) thresholds and linear fits of stimulation level across all 29 volunteers at isocenter: male (*square*), female (*circle*), age > 50 years (*dashed line*), age < 50 years (*solid line*). No value or linear fit is plotted for volunteers who did not report any stimulation; however, they were included in the population threshold calculation.
**Figure S4.** Individual plots of gradient amplitude (mT/m) thresholds (*blue*) with respect to age (years) at different rise times (μs). Volunteers who did not report any stimulation at a given rise time are assumed conservatively to be 1 mT/m higher than the maximum accessible gradient amplitude (e.g., impulse gradient coil can achieve maximum 90 mT/m for 0.1‐ms rise time) and are plotted in red. The linear trendline (*yellow*) thereby accounts for the volunteers who felt no peripheral nerve stimulation (PNS).
**Table S1.** Peripheral nerve stimulation (PNS) thresholds (zero‐to‐peak, in mT/m) for individual subjects from the Berkeley data, per rise time per gradient axis, up to system hardware limits at 2 cm offset along the z‐axis. Subjects who experienced no PNS for that gradient axis and rise time are marked “No PNS.” Subjects who were not tested on that gradient axis are marked with a “–.”
**Table S2.** Peripheral nerve stimulation (PNS) thresholds (zero‐to‐peak, in mT/m) for individual subjects from the Berkeley data, per rise time per gradient axis, up to system hardware limits at 4 cm offset along the z‐axis. Subjects who experienced no PNS for that gradient axis and rise time are marked “No PNS.” Subjects who were not tested on that gradient axis are marked with a “–.”
**Table S3.** Coefficients $\beta_{n}$ obtained by the logistic regression fit for the different data sets and axes, when considering risetime τ, 0‐peak gradient amplitude Δ G, age, age τ, gender, and gender τ as model‐parameters. *p*‐Values are indicated when statistically significant.
**Table S4.** Coefficients $\beta_{n}$ obtained by the logistic regression fit for the different data sets and axes, when considering risetime τ, 0‐peak gradient amplitude Δ G, age, age τ, gender, gender τ, z‐offset, and z‐offset τ as model‐parameters. *p*‐Values are indicated when statistically significant.
