# Supplementary material for: Reassessment of peripheral nerve stimulation thresholds for the Impulse model‐optimized asymmetric head gradient coil
Source: Magn Reson Med. 2025 May 23;94(3):1326–38. doi: 10.1002/mrm.30523 (PMC12202733; doi:10.1002/mrm.30523)
Supplement: Supplementary file 1 — Figure S1. The y‐axis and z‐axis simulated thresholds with three directions of displacement from modeling. Figure S2. Nonlinearity distortions with 15.6‐cm‐diameter agar spherical phantom. Top: The different localizer sagittal images (0.5 mm in‐plane resolution, 3‐mm thickness) acquired at isocenter (0 cm), 2, 4 and 6 cm toward feet are shown without (left) and with (right) distortion corrections. Bottom: Same as top but with the images translated by the respective displacements and superposed to the one located at isocenter. Figure S3. Individual peripheral nerve stimulation (PNS) thresholds and linear fits of stimulation level across all 29 volunteers at isocenter: male (square), female (circle), age > 50 years (dashed line), age < 50 years (solid line). No value or linear fit is plotted for volunteers who did not report any stimulation; however, they were included in the population threshold calculation. Figure S4. Individual plots of gradient amplitude (mT/m) thresholds (blue) with respect to age (years) at different rise times (μs). Volunteers who did not report any stimulation at a given rise time are assumed conservatively to be 1 mT/m higher than the maximum accessible gradient amplitude (e.g., impulse gradient coil can achieve maximum 90 mT/m for 0.1‐ms rise time) and are plotted in red. The linear trendline (yellow) thereby accounts for the volunteers who felt no peripheral nerve stimulation (PNS). Table S1. Peripheral nerve stimulation (PNS) thresholds (zero‐to‐peak, in mT/m) for individual subjects from the Berkeley data, per rise time per gradient axis, up to system hardware limits at 2 cm offset along the z‐axis. Subjects who experienced no PNS for that gradient axis and rise time are marked “No PNS.” Subjects who were not tested on that gradient axis are marked with a “–.” Table S2. Peripheral nerve stimulation (PNS) thresholds (zero‐to‐peak, in mT/m) for individual subjects from the Berkeley data, per rise time per gradient axis, up to system hardwar [file MRM-94-1326-s001.docx]

**Supplemental Material**

**Reassessment of PNS thresholds for the Impulse model-optimized asymmetric head gradient coil**

David A. Feinberg*, Samantha J. Ma, Erica Walker, Alexander J. S. Beckett, Dominik Rattenbacher, Elmar Rummert, Peter Dietz, Mathias Davids, Nicolas Boulant

*Corresponding Author: David A. Feinberg

Brain Imaging Center, Helen Wills Neuroscience Institute, Department of Neuroscience, University of California, Berkeley, CA, USA

Email: [david.feinberg@berkeley.edu](mailto:david.feinberg@berkeley.edu)


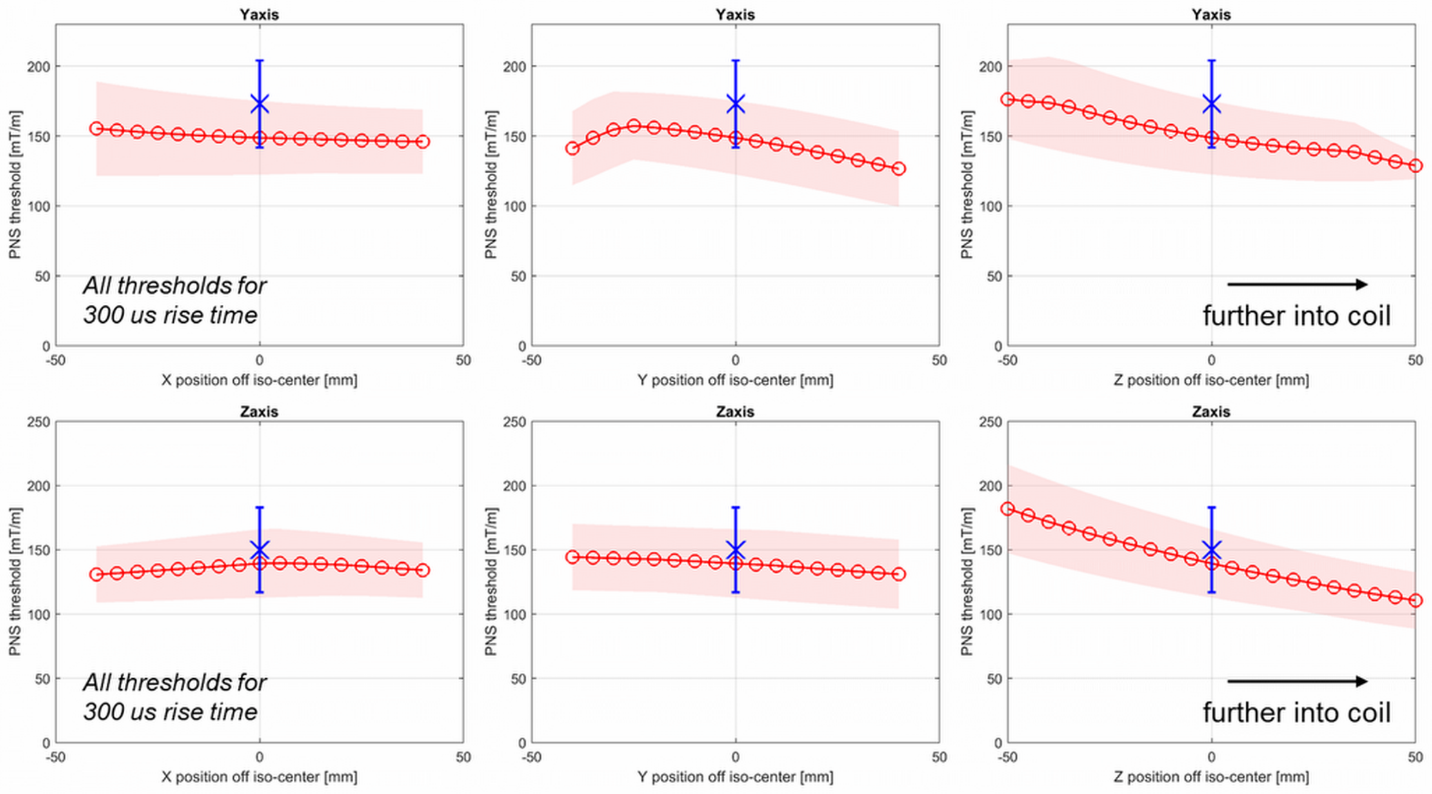


Figure S1. Y-axis and Z-axis simulated thresholds with three directions of displacement from modeling.


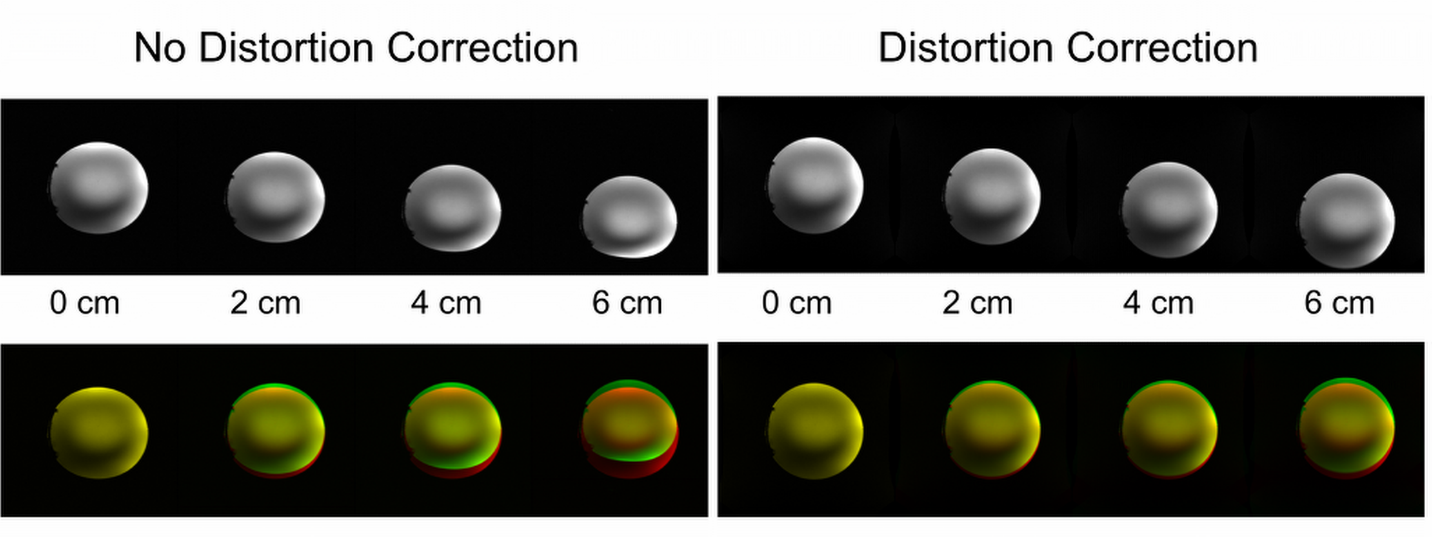


Figure S2. Nonlinearity distortions with 15.6 cm diameter agar spherical phantom. Top: the different localizer sagittal images (0.5 mm in plane resolution, 3 mm thickness) acquired at isocenter (0 cm), 2 cm, 4 cm and 6 cm towards feet are shown without (left) and with (right) distortion corrections. Bottom: same as top but with the images translated by the respective displacements and superposed to the one located at isocenter.


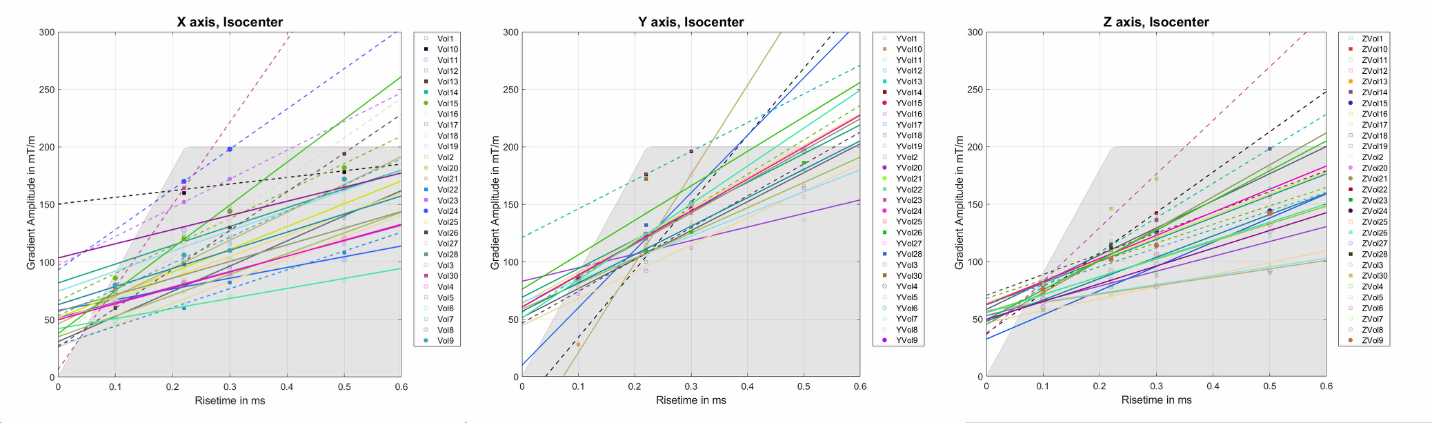


Figure S3. Individual PNS thresholds and linear fits of stimulation level across all 29 volunteers at isocenter. Male (square), Female (circle), Age > 50 years (dashed line), Age < 50 years (solid line). No value or linear fit is plotted for volunteers who did not report any stimulation; however, they were included in the population threshold calculation.


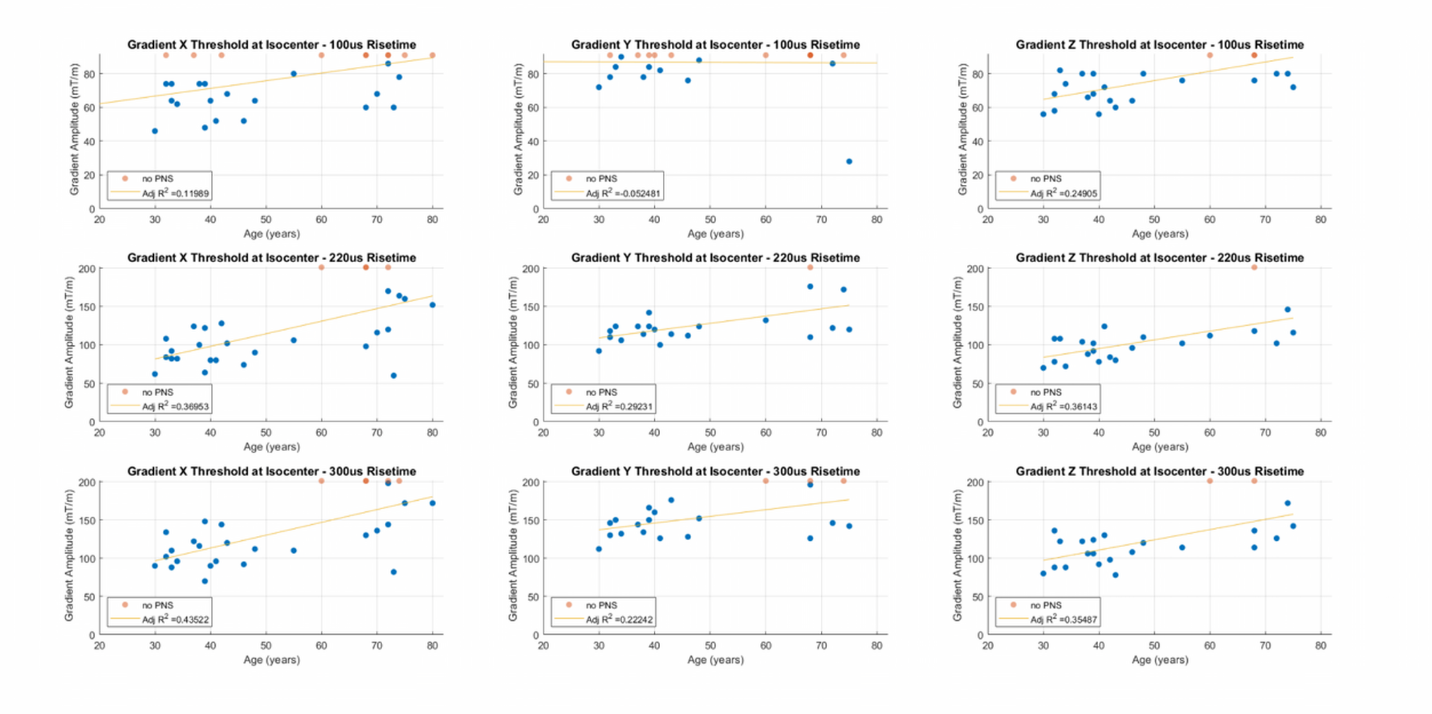


Figure S4. Individual plots of gradient amplitude (mT/m) thresholds (blue) with respect to age (years) at different rise times (μs). Volunteers who did not report any stimulation at a given rise time are assumed conservatively to be 1 mT/m higher than the maximum accessible gradient amplitude (e.g. Impulse gradient coil can achieve maximum 90 mT/m for 0.1 ms rise time) and plotted in red. The linear trendline (yellow) thereby accounts for the volunteers who felt no PNS.

Berkeley (2cm Offset)

|  |  | | | | GX | Rise times (us) | | | | GY | Rise times (us) | | | | GZ | Rise times (us) | | | |
| --- | --- | --- | --- | --- | --- | --- | --- | --- | --- | --- | --- | --- | --- | --- | --- | --- | --- | --- | --- |
| **Subject** | **Sex** | **Age (year)** | **Height (cm)** | **Weight**  **(kg)** | **Location** | **100** | **220** | **300** | **500** | **Location** | **100** | **220** | **300** | **500** | **Location** | **100** | **220** | **300** | **500** |
| 1 | M | 34 | 172 | 73.5 | Forehead/Nose | 40 | 62 | 84 | 180 | Shoulder | 72 | 124 | 148 | 138 | Upper chest | 80 | 128 | 132 | 192 |
| 2 | M | 46 | 177 | 80 | Forehead | 58 | 84 | 108 | 128 | Chest/Arms | 82 | 106 | 128 | 166 | Upper Chest | 68 | 88 | 104 | 144 |
| 3 | F | 32 | 175 | 68 | Forehead/Nose | 86 | 120 | 142 | 182 | Arms | No PNS | 140 | 156 | No PNS | Chest | 80 | 118 | 148 | 198 |
| 4 | F | 33 | 160 | 63 | Nose | 84 | 104 | 128 | 152 | Arms | 92 | 128 | 150 | No PNS | Upper Chest | 80 | 108 | 144 | No PNS |
| 5 | M | 38 | 178 | 79 | Nose | 86 | 152 | 198 | No PNS | Nose/Shoulders | 90 | 142 | 168 | No PNS | Chest/Shoulders | 86 | 110 | 148 | 188 |
| 6 | F | 39 | 172 | 100 | Nose | 58 | 84 | 92 | 122 | Shoulders | 54 | 86 | 114 | 182 | Chest/Shoulders | 70 | 96 | 110 | 162 |
| 7 | M | 39 | 172 | 70 | Nose | No PNS | 124 | 156 | No PNS | Shoulders | 82 | 112 | 128 | 178 | Shoulders | 82 | 118 | 150 | No PNS |
| 8 | F | 42 | 172 | 72 | - | - | - | - | - | - | - | - | - | - | - | - | - | - | - |
| 9 | F | 55 | 165 | 70 | - | - | - | - | - | - | - | - | - | - | - | - | - | - | - |
| 10 | M | 75 | 185.4 | 74.8 | Nose/Forehead | No PNS | 138 | 198 | No PNS | Shoulders | No PNS | 126 | 140 | No PNS | Shoulders | No PNS | 120 | 152 | No PNS |
| 11 | F | 40 | 170.18 | 86.18 | Nose | 86 | 106 | 130 | 148 | Neck/Shoulders | 82 | 114 | 128 | 150 | Armpit | 54 | 66 | 86 | 110 |
| 12 | M | 37 | 185 | 84 | Forehead | No PNS | 174 | No PNS | No PNS | Shoulders/Hands | No PNS | 132 | 162 | No PNS | Chest | 94 | 122 | 140 | 180 |
| 13 | M | 68 | 169 | 75 | - | - | - | - | - | - | - | - | - | - | - | - | - | - | - |
| 14 | M | 68 | 180.3 | 97 | - | - | - | - | - | - | - | - | - | - | - | - | - | - | - |
| 15 | F | 72 | 157.48 | 52.6 | Forehead/Nose | No PNS | 172 | No PNS | No PNS | N/A | No PNS | No PNS | No PNS | No PNS | - | - | - | - | - |
| 16 | M | 30 | 184 | 89 |  | 66 | 76 | 112 | 160 |  | 78 | 108 | 124 | 182 |  | 62 | 78 | 86 | 98 |
| 17 | M | 41 | 183 | 83 | - | - | - | - | - | - | - | - | - | - | - | - | - | - | - |
| 18 | M | 48 | 193 | 95 | - | - | - | - | - | - | - | - | - | - | - | - | - | - | - |
| 19 | M | 32 | 160 | 68 | Nose | No PNS | 150 | 152 | No PNS | - | - | - | - | - | - | - | - | - | - |
| 20 | F | 72 | 178 | 81.65 | N/A | No PNS | No PNS | No PNS | No PNS | N/A | No PNS | No PNS | No PNS | No PNS |  | No PNS | 140 | 168 | No PNS |
| 21 | F | 70 | 152 | 52.2 | Eyebrow | 78 | 138 | 180 | No PNS | N/A | No PNS | No PNS | No PNS | No PNS | N/A | No PNS | No PNS | No PNS | No PNS |
| 22 | M | 73 | 178 | 74.8 | Forehead/Nose | 82 | 102 | 116 | 144 |  | 64 | 78 | 82 | 104 |  | 48 | 66 | 72 | 106 |
| 23 | M | 80 | 177 | 74.84 | N/A | No PNS | No PNS | No PNS | No PNS | N/A | No PNS | No PNS | No PNS | No PNS |  | No PNS | 148 | 166 | No PNS |
| 24 | F | 72 | 157 | 51.26 | N/A | No PNS | No PNS | No PNS | No PNS | N/A | No PNS | No PNS | No PNS | No PNS |  | No PNS | 142 | 174 | No PNS |
| 25 | M | 33 | 160 | 54 | Nose | 86 | 134 | 152 | 192 |  | 88 | 126 | 136 | 174 |  | 82 | 106 | 134 | 166 |
| 26 | M | 68 |  |  | Nose | No PNS | 130 | 166 | No PNS | - | - | - | - | - | - | - | - | - | - |
| 27 | F | 43 | 165 | 54.43 | Nose | 86 | 138 | 166 | No PNS | - | - | - | - | - | - | - | - | - | - |
| 28 | M | 60 | 172 | 90.71 | N/A | No PNS | No PNS | No PNS | No PNS | - | - | - | - | - | - | - | - | - | - |
| 29 | M | 74 | 176 | 58 | Head | No PNS | 198 | No PNS | No PNS | - | - | - | - | - | - | - | - | - | - |

Table S1 – PNS Thresholds (zero-to-peak, in mT/m) for individual subjects from the Berkeley data, per rise time per gradient axis, up to system hardware limits at 2cm offset along the Z-axis. Subjects who experienced no PNS for that gradient axis and rise time are marked ‘No PNS’. Subjects who were not tested on that gradient axis are marked with a ‘ - ‘.

Berkeley (4cm Offset)

|  |  | | | | GX | Rise times (us) | | | | GY | Rise times (us) | | | | GZ | Rise times (us) | | | |
| --- | --- | --- | --- | --- | --- | --- | --- | --- | --- | --- | --- | --- | --- | --- | --- | --- | --- | --- | --- |
| **Subject** | **Sex** | **Age (year)** | **Height (cm)** | **Weight**  **(kg)** | **Location** | **100** | **220** | **300** | **500** | **Location** | **100** | **220** | **300** | **500** | **Location** | **100** | **220** | **300** | **500** |
| 1 | M | 34 | 172 | 73.5 | Forehead/Nose | 86 | 160 | No PNS | No PNS | Shoulder | No PNS | 120 | No PNS | No PNS | Upper chest | 72 | 116 | 122 | 150 |
| 2 | M | 46 | 177 | 80 | Forehead | No PNS | 152 | 186 | No PNS | Chest/Arms | 74 | 92 | 102 | 138 | Upper Chest | 68 | 100 | 112 | 154 |
| 3 | F | 32 | 175 | 68 | Forehead/Nose | No PNS | 152 | 196 | No PNS | Arms | No PNS | 136 | 150 | No PNS | Chest | 72 | 114 | 156 | No PNS |
| 4 | F | 33 | 160 | 63 | Nose | 80 | 108 | 128 | 158 | Arms | 84 | 108 | 132 | No PNS | Upper Chest | 68 | 108 | 144 | No PNS |
| 5 | M | 38 | 178 | 79 | Nose | No PNS | 140 | No PNS | No PNS | Nose/Shoulders | 86 | 124 | 148 | No PNS | Chest/Shoulders | 84 | 126 | 146 | 196 |
| 6 | F | 39 | 172 | 100 | Nose | 86 | 118 | 136 | 180 | Shoulders | 74 | 100 | 114 | 176 | Chest/Shoulders | 74 | 104 | 116 | 158 |
| 7 | M | 39 | 172 | 70 | Nose | No PNS | 168 | 192 | No PNS | Shoulders | 86 | 118 | 148 | 194 | Shoulders | 82 | 124 | 144 | 192 |
| 8 | F | 42 | 172 | 72 | - | - | - | - | - | - | - | - | - | - | - | - | - | - | - |
| 9 | F | 55 | 165 | 70 | - | - | - | - | - | - | - | - | - | - | - | - | - | - | - |
| 10 | M | 75 | 185.4 | 74.8 | Nose/Forehead | No PNS | 180 | No PNS | No PNS | Shoulders | No PNS | 124 | 150 | No PNS | Shoulders | No PNS | 152 | 180 | No PNS |
| 11 | F | 40 | 170.18 | 86.18 | - | - | - | - | - | - | - | - | - | - | - | - | - | - | - |
| 12 | M | 37 | 185 | 84 | Forehead | No PNS | 178 | No PNS | No PNS | Shoulders/Hands | No PNS | 114 | 142 | 178 | Chest | 88 | 114 | 132 | 172 |
| 13 | M | 68 | 169 | 75 | - | - | - | - | - | - | - | - | - | - | - | - | - | - | - |
| 14 | M | 68 | 180.3 | 97 | - | - | - | - | - | - | - | - | - | - | - | - | - | - | - |
| 15 | F | 72 | 157.48 | 52.6 | - | - | - | - | - | - | - | - | - | - | - | - | - | - | - |
| 16 | M | 30 | 184 | 89 |  | - | - | - | - |  | - | - | - | - |  | - | - | - | - |
| 17 | M | 41 | 183 | 83 | - | - | - | - | - | - | - | - | - | - | - | - | - | - | - |
| 18 | M | 48 | 193 | 95 | - | - | - | - | - | - | - | - | - | - | - | - | - | - | - |
| 19 | M | 32 | 160 | 68 | N/A | No PNS | No PNS | No PNS | No PNS | - | - | - | - | - | - | - | - | - | - |
| 20 | F | 72 | 178 | 81.65 | N/A | No PNS | No PNS | No PNS | No PNS | N/A | No PNS | No PNS | No PNS | No PNS |  | No PNS | 156 | No PNS | No PNS |
| 21 | F | 70 | 152 | 52.2 | N/A | No PNS | No PNS | No PNS | No PNS | - | - | - | - | - | - | - | - | - | - |
| 22 | M | 73 | 178 | 74.8 | Forehead/Nose | 82 | 142 | 134 | 198 |  | 66 | 80 | 88 | 108 |  | 72 | 92 | 104 | 126 |
| 23 | M | 80 | 177 | 74.84 | N/A | No PNS | No PNS | No PNS | No PNS | - | - | - | - | - |  | No PNS | 136 | 166 | 194 |
| 24 | F | 72 | 157 | 51.26 | N/A | No PNS | No PNS | No PNS | No PNS | N/A | No PNS | No PNS | No PNS | No PNS |  | No PNS | 152 | 196 | No PNS |
| 25 | M | 33 | 160 | 54 | Nose | No PNS | 120 | No PNS | No PNS |  | 72 | 98 | 138 | 180 |  | 86 | 118 | 130 | 178 |
| 26 | M | 68 |  |  | N/A | No PNS | No PNS | No PNS | No PNS | - | - | - | - | - | - | - | - | - | - |
| 27 | F | 43 | 165 | 54.43 | N/A | No PNS | No PNS | No PNS | No PNS | - | - | - | - | - | - | - | - | - | - |
| 28 | M | 60 | 172 | 90.71 | - | - | - | - | - | - | - | - | - | - | - | - | - | - | - |
| 29 | M | 74 | 176 | 58 | N/A | No PNS | No PNS | No PNS | No PNS | - | - | - | - | - | - | - | - | - | - |

Table S2 – PNS Thresholds (zero-to-peak, in mT/m) for individual subjects from the Berkeley data, per rise time per gradient axis, up to system hardware limits at 4cm offset along the Z-axis. Subjects who experienced no PNS for that gradient axis and rise time are marked ‘No PNS’. Subjects who were not tested on that gradient axis are marked with a ‘ - ‘.

| Parameter | Intercept | $\tau$ | $\Delta$G | Age | Age $\cdot\tau$ | Gender | Gender $\cdot\tau$ |
| --- | --- | --- | --- | --- | --- | --- | --- |
| Coefficient | $\beta_{0}$ | $\beta_{1} \left( \frac{1}{ms} \right)$ | $\beta_{2} \left( \frac{m}{mT} \right)$ | $\beta_{3}$ $\left( \frac{1}{a} \right)$ | $\beta_{4}$ $\left( \frac{1}{a\cdot ms} \right)$ | $\beta_{5}$ | $\beta_{6}$ $\left( \frac{1}{ms} \right)$ |
| X-axis UCB | -2.9859 ± 0.2243 (P<0.01) | -13.5905 ± 0.7114 (P<0.01) | +0.0551 ± 0.0024 (P<0.01) | -0.0234 ± 0.0096 (P<0.05) | -0.2177 ± 0.0348 (P<0.01) | +0.0491 ± 0.1560 | -0.7864 ± 0.5257 |
| Y-axis UCB | -4.5437 ± 0.4002 (P<0.01) | -28.1611 ± 1.8395 (P<0.01) | +0.0862 ± 0.0050 (P<0.01) | +0.0507 ± 0.0189 (P<0.01) | -0.4991 ± 0.0803 (P<0.01) | -0.6973 ± 0.2980 (P<0.05) | +4.0217 ± 1.1518 (P<0.01) |
| Z-axis UCB | -4.0133 ± 0.2694 (P<0.01) | -15.9895 ± 0.8772 (P<0.01) | +0.0751 ± 0.0035 (P<0.01) | -0.0255 ± 0.0118 (P<0.05) | -0.1960 ± 0.0406 (P<0.01) | -0.2226 ± 0.1823 | -0.4660 ± 0.5926 |
| X-axis ERL | -2.5309 ± 0.3438 (P<0.01) | -18.0558 ± 1.2759 (P<0.01) | +0.0414 ± 0.0027 (P<0.01) | +0.0137 ± 0.0192 | -0.1318 ± 0.0799 | -0.1499 ± 0.2661 | +1.5317 ± 1.1395 |
| Y-axis ERL | -4.1071 ± 0.3054 (P<0.01) | -18.3278 ± 1.0319 (P<0.01) | +0.0578 ± 0.0027 (P<0.01) | -0.0174 ± 0.0162 | -0.0888 ± 0.0603 | +0.2911 ± 0.2208 | -0.7185 ± 0.8444 |
| Z-axis ERL | -4.2010 ± 0.2595 (P<0.01) | -16.9043 ± 0.8866 (P<0.01) | +0.0635 ± 0.0026 (P<0.01) | -0.0236 ± 0.0144 | -0.0822 ± 0.0517 | +0.2468 ± 0.1920 | +0.4459 ± 0.6943 |
| X-axis ERL+UCB | -2.4671 ± 0.1733 (P<0.01) | -13.1717 ± 0.5714 (P<0.01) | +0.0408 ± 0.0015 (P<0.01) | -0.0009 ± 0.0077 | -0.2266 ± 0.0296 (P<0.01) | +0.1123 ± 0.1189 | +0.4765 ± 0.4495 |
| Y-axis ERL+UCB | -4.2476 ± 0.2422 (P<0.01) | -20.0021 ± 0.8744 (P<0.01) | +0.0646 ± 0.0023 (P<0.01) | -0.0058 ± 0.0104 | -0.1865 ± 0.0404 (P<0.01) | +0.0736 ± 0.1610 | +0.5806 ± 0.6257 |
| Z-axis ERL+UCB | -4.0532 ± 0.1808 (P<0.01) | -15.0115 ± 0.5538 (P<0.01) | +0.0646 ± 0.0020 (P<0.01) | -0.0289 ± 0.0079 (P<0.01) | -0.1450 ± 0.0269 (P<0.01) | +0.1596 ± 0.1163 | +0.2123 ± 0.3961 |

Table S3. Coefficients $\beta_{n}$ obtained by the logistic regression fit for the different data sets and axes, when considering risetime $\tau,$ 0-peak gradient amplitude $\Delta$G, age, age$\cdot\tau$, gender and gender$\cdot\tau$ as model-parameters. P-values are indicated when statistically significant.

| Parameter | Intercept | $\tau$ | $\Delta$G | Age | Age $\cdot\tau$ | Gender | Gender $\cdot\tau$ | Offset | Offset $\cdot\tau$ |
| --- | --- | --- | --- | --- | --- | --- | --- | --- | --- |
| Coefficient | $\beta_{0}$ | $\beta_{1}$ $\left( \frac{1}{ms} \right)$ | $\beta_{2}$ $\left( \frac{m}{mT} \right)$ | $\beta_{3}$ $\left( \frac{1}{a} \right)$ | $\beta_{4}$ $\left( \frac{1}{a\cdot ms} \right)$ | $\beta_{5}$ | $\beta_{6}$ $\left( \frac{1}{ms} \right)$ | $\beta_{7}$ $\left( \frac{1}{cm} \right)$ | $\beta_{8}$ $\left( \frac{1}{cm\cdot ms} \right)$ |
| X-axis UCB | -2.7535 ± 0.2093 (P<0.01) | -13.171 ± 0.6987 (P<0.01) | +0.0524 ± 0.0018 (P<0.01) | +0.0052 ± 0.0073 | -0.2896 ± 0.0306 (P<0.01) | +0.2043 ± 0.1219 | -1.5198 ± 0.4608 (P<0.01) | +0.1269 ± 0.0794 | -3.5802 ± 0.3363 (P<0.01) |
| Y-axis UCB | -3.6124 ± 0.2677 (P<0.01) | -20.259 ± 1.0063 (P<0.01) | +0.0637 ± 0.0023 (P<0.01) | -0.0185 ± 0.0089 (P<0.05) | -0.0950 ± 0.0347 (P<0.01) | -0.1876 ± 0.1591 | +2.9357 ± 0.6275 (P<0.01) | -0.0349 ± 0.0894 | +0.5011 ± 0.3303 |
| Z-axis UCB | -3.5129 ± 0.2054 (P<0.01) | -14.388 ± 0.6566 (P<0.01) | +0.0661 ± 0.0020 (P<0.01) | -0.0225 ± 0.0069 (P<0.01) | -0.1111 ± 0.0247 (P<0.01) | -0.1318 ± 0.1181 | +0.8416 ± 0.4019 (P<0.05) | -0.0268 ± 0.0706 | -1.1490 ± 0.2444 (P<0.01) |

Table S4. Coefficients $\beta_{n}$ obtained by the logistic regression fit for the different data sets and axes, when considering risetime $\tau,$ 0-peak gradient amplitude $\Delta$G, age, age$\cdot\tau$, gender, gender$\cdot\tau$, z-offset and z-offset$\cdot\tau$ as model-parameters. P-values are indicated when statistically significant.
